# Determination of α lamellae orientation in a β-Ti alloy using electron backscatter diffraction

**DOI:** 10.1107/S160057672400400X

**Published:** 2024-06-27

**Authors:** Petr Harcuba, Jana Šmilauerová, Miloš Janeček, Jan Ilavský, Václav Holý

**Affiliations:** a Department of Physics of Materials, Faculty of Mathematics and Physics, Charles University, Ke Karlovu 5, 121 16Prague 2, Czech Republic; b X-ray Science Division, Advanced Photon Source, Argonne National Laboratory, Argonne, IL60439, USA; c Department of Condensed Matter Physics, Faculty of Mathematics and Physics, Charles University, Ke Karlovu 5, 121 16Prague 2, Czech Republic; d Department of Condensed Matter Physics, Faculty of Science, Masaryk University, Kotlářská 2, 611 37Brno, Czech Republic; Montanuniversität Leoben, Austria

**Keywords:** metastable titanium alloys, α phase, habit planes, orientation relationships

## Abstract

The spatial orientation of α lamellae in a metastable β-Ti matrix of Timetal LCB (Ti–6.8 Mo–4.5 Fe–1.5 Al in wt%) was examined and the orientation of the hexagonal close-packed α lattice in the α lamella was determined. For this purpose, a combination of methods of small-angle X-ray scattering, scanning electron microscopy and electron backscatter diffraction was used. The habit planes of α laths are close to {111}_β_, which corresponds to (1320)_α_ in the hexagonal coordinate system of the α phase. The longest α lamella direction lies approximately along one of the 〈110〉_β_ directions which are parallel to the specific habit plane. Taking into account the average lattice parameters of the β and α phases in aged conditions in Timetal LCB, it was possible to index all main axes and faces of an α lath not only in the cubic coordinate system of the parent β phase but also in the hexagonal system of the α phase.

## Abstract 2

A combination of electron backscatter diffraction and small-angle X-ray scattering analyses was employed to index all main axes and faces of an α lath not only in the cubic coordinate system of the parent β phase but also in the hexagonal system of the α phase.

## 1. Introduction

Titanium is an allotropic material which undergoes a phase transition at 882°C from a low-temperature α phase [hexagonal close-packed (h.c.p.) structure] to a high-temperature β phase [body-centred cubic (b.c.c.)] (Lütjering & Williams, 2007 ▸). With increasing additions of alloying elements which stabilize the β phase at lower temperatures, it is possible to achieve such a composition of the alloy that the martensitic transformation to the α′ phase is suppressed and the β phase is retained in a metastable state after rapid cooling to room temperature (Leyens & Peters, 2003 ▸). These metastable β-titanium alloys are very versatile materials due to the combination of good corrosion resistance, low density and high strength achievable through ageing treatment (Ankem & Greene, 1999 ▸). When exposed to elevated temperatures, metastable β-Ti alloys exhibit complex phase transformations, the nature of which strongly depends on the treatment conditions (*e.g.* ageing temperature and time, previous treatments *etc*.) and the specific alloy composition. At lower ageing temperatures, either the metastable β phase decomposes into β′ + β or particles of the ω phase form in the material (Kolli & Devaraj, 2018 ▸). Eventually, these ‘chemical inhomogeneities’ which are depleted in β-stabilizing elements (*i.e.* β′ or ω) serve as precursors for precipitation of very fine, uniformly distributed lamellae of the α phase (Santhosh *et al.*, 2017 ▸; Nag *et al.*, 2009*b* ▸). On the other hand, isothermal ageing at higher temperatures results in α lamellae forming directly from the β matrix and consequently exhibiting a coarser, elongated morphology (Ankem & Seagle, 1984 ▸).

Between the h.c.p. α and the b.c.c. β, the Burgers orientation relationship (OR) has been reported (Burgers, 1934 ▸): 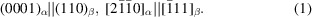
Equation (1) is written for one possible mutual orientation of the β and α lattices. Since there are six different {110} planes in a cubic lattice and each contains two directions of the type 〈111〉, the Burgers OR yields 12 different crystallographic variants of the α phase with respect to the β matrix.

However, the possibility of α phase formation according to the Potter OR (Pitsch & Schrader, 1958 ▸), *i.e.*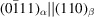, 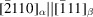, was suggested for intragranular α precipitates in Ti–15 V–3 Cr–3 Sn–3 Al exhibiting a triangular arrangement (Miyano *et al.*, 2002 ▸, 2006 ▸) and for α precipitates in Ti–40 Mo (Furuhara *et al.*, 1998 ▸). A Potter OR was also identified between grain boundary α precipitates and one of the adjacent β grains in Ti–22 V–4 Al alloy using Kikuchi pattern analysis (Fujiwara *et al.*, 1998 ▸). The difference between Burgers and Potter ORs is a simple rotation by ∼2° around 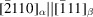; however, a correlation between the specific OR and the shape of an α embryo was observed (Miyano *et al.*, 2002 ▸).

In the present study, we consider the metastable β-Ti alloy Ti–6.8 Mo–4.5 Fe–1.5 Al (in wt%), for which we assume the Burgers OR is valid. This assumption is supported by the literature [see *e.g.* Sakamoto *et al.* (2007 ▸), Azimzadeh & Rack (1998 ▸) and Lenain *et al.* (2005 ▸)].

The mutual crystallographic orientation between α and β lattices (*i.e.* the Burgers/Potter OR) does not tell us anything about the spatial orientation of the α lamella itself in the β matrix. This spatial orientation, *i.e.* the indices of the α lamella broad face (habit plane) and those of the two smaller faces, is not a fully resolved topic and there is much inconsistency in the literature. The α lath spatial orientation has been investigated for several alloy systems. While some research determined that the habit plane of an α lamella is (approximately) parallel to one of the {111}_β_ planes (Furuhara *et al.*, 1991 ▸, 1998 ▸; Guo *et al.*, 2010 ▸; Ohmori *et al.*, 1998 ▸), other work has reported the habit plane close to 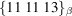 (Shi *et al.*, 2012 ▸; Furuhara *et al.*, 1996 ▸; Zherebtsov *et al.*, 2010 ▸; Bhattacharyya *et al.*, 2007 ▸), or {334} for an h.c.p. α′ martensite (Qiu *et al.*, 2014 ▸). A transmission Kikuchi diffraction study analysed the α variants and their habit planes, while considering 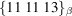 and {112}_β_ as two possible options for the orientation of the habit plane (Tong *et al.*, 2017 ▸).

Although the present paper focuses mainly on the investigation of α lamellae by scanning electron microscopy (SEM) and electron backscatter diffraction (EBSD), we wanted to directly link its results to the small-angle X-ray scattering (SAXS) analysis carried out in our previous paper (Šmilauerová *et al.*, 2023 ▸). Therefore, in this article we show selected SAXS patterns arising from the α lamellae and we compare our SEM observations with a simulation of the α microstructure calculated using the results of SAXS data fitting (Šmilauerová *et al.*, 2023 ▸). This comparison validates our previous SAXS results and demonstrates the complementarity of SEM/EBSD and SAXS techniques. While SAXS is an indirect method, in which the incident X-ray beam is scattered by inhomogeneities in electron density within the sample (Pauw, 2013 ▸; Fratzl, 2003 ▸), SEM provides a direct insight into the microstructure of precipitates and their spatial arrangement. As opposed to the local character of SEM micrographs, SAXS allows us to obtain statistically relevant information from a significantly larger volume of the sample and, consequently, from many different α lamellae. Note also that SAXS is not sensitive to the crystallography but only to the contrast in electronic density, which in this case corresponds to the chemical difference between the α and β phases. On the other hand, EBSD is able to provide us with crystallographic information, including the mutual orientations of α platelets and the β matrix.

## 2. Material and experimental methods

This study was carried out on a single crystal of Ti–6.8 Mo–4.5 Fe–1.5 Al (wt%) alloy, also known as LCB (low-cost beta). The composition of this alloy classifies it as a metastable β-Ti alloy. The single crystal was grown using a floating zone technique; details of the growth of the single crystal and the characterization of the resulting ingot have been reported by Šmilauerová *et al.* (2014 ▸). The ingot was solution treated at 860°C for 4 h in an evacuated quartz tube and quenched in water. This treatment ensured homogenization of the ingot and yielded an initial condition with the β phase retained in a thermodynamically metastable state.

Subsequently, disc-shaped samples were cut from the ingot and a series of different conditions were prepared by ageing in a temperature range in which α phase precipitates in the material [for details, see Šmilauerová *et al.* (2023 ▸)]. For the purpose of demonstrating the α platelets’ orientation, a sample aged at 540°C for 16 h was selected for the present study. In these conditions, the α lamellae were large enough to be reliably characterized by SEM, but their size did not exceed the resolution limit of the SAXS method.

The orientation of the single crystal was determined using the Laue X-ray backscattering method (Photonic Science system equipped with a W X-ray tube). The diffractograms were indexed using the *OrientExpress* software (Laugier & Bochu, 2005 ▸). A stereographic projection of the β matrix orientation shown in this paper was produced from *Orient­Express* data using MATLAB software (The MathWorks Inc., 2019 ▸).

SAXS was performed at a dedicated SAXS beamline at the Advanced Photon Source, Argonne National Laboratory, USA (Ilavsky *et al.*, 2009 ▸). The incident photon energy was 25 keV and the beam size at the sample was 150 × 150 µm. The scattered intensity was detected by a large 2D detector with 2048 × 2048 pixels (Mar165). The incident as well as the scattered beam passed through evacuated tubes to minimize air scattering. The sample was mounted on a goniometer, which allowed us to align the crystallographic axes with respect to the laboratory coordinates. The single-crystalline sample was measured in three orientations: [001], [110] and [111] directions in the β matrix being parallel to the primary beam. The obtained SAXS data were calibrated using Ag behenate and glassy carbon samples for angular and intensity calibrations, respectively (Šmilauerová *et al.*, 2014 ▸).

In our previous paper (Šmilauerová *et al.*, 2023 ▸) we used the SAXS data for the determination of the mean lamella size and orientation, while assuming the shape of the α lamellae to be a triaxial ellipsoid. In the present paper, we used the fitted lamella parameters (*i.e.* the mean values of the ellipsoid half-axes *R*_*a*_ < *R*_*b*_ < *R*_*c*_, the angles defining its orientation and the order of the gamma distribution of the mean size) to simulate the α lamella microstructure. The simulation was calculated for the same orientation of the sample surface as that of the SEM images shown in this paper. In the simulation we generated random positions of the lamella centres and random lamella sizes, keeping fixed the ratios *R*_*b*_/*R*_*a*_ and *R*_*c*_/*R*_*a*_. Each lamella was then subjected to a randomly chosen rotation symmetry operation, which belongs to the full cubic point group *O*_*h*_. In the simulation, we used the obvious constraint which forbids the intersection of the lamellae.

The SEM experiments were performed using a Zeiss Auriga Compact microscope equipped with a field emission gun, a focused ion beam (FIB) and analytical detectors including an EBSD camera. SEM images of the α lamella morphology were taken using a Thermo Fisher Scientific Apreo 2S. EBSD data were cleaned using one iteration of grain dilation; fewer than 5% of the measured points were changed during the cleaning procedure.

## 3. Results and discussion

**Figure 1:**
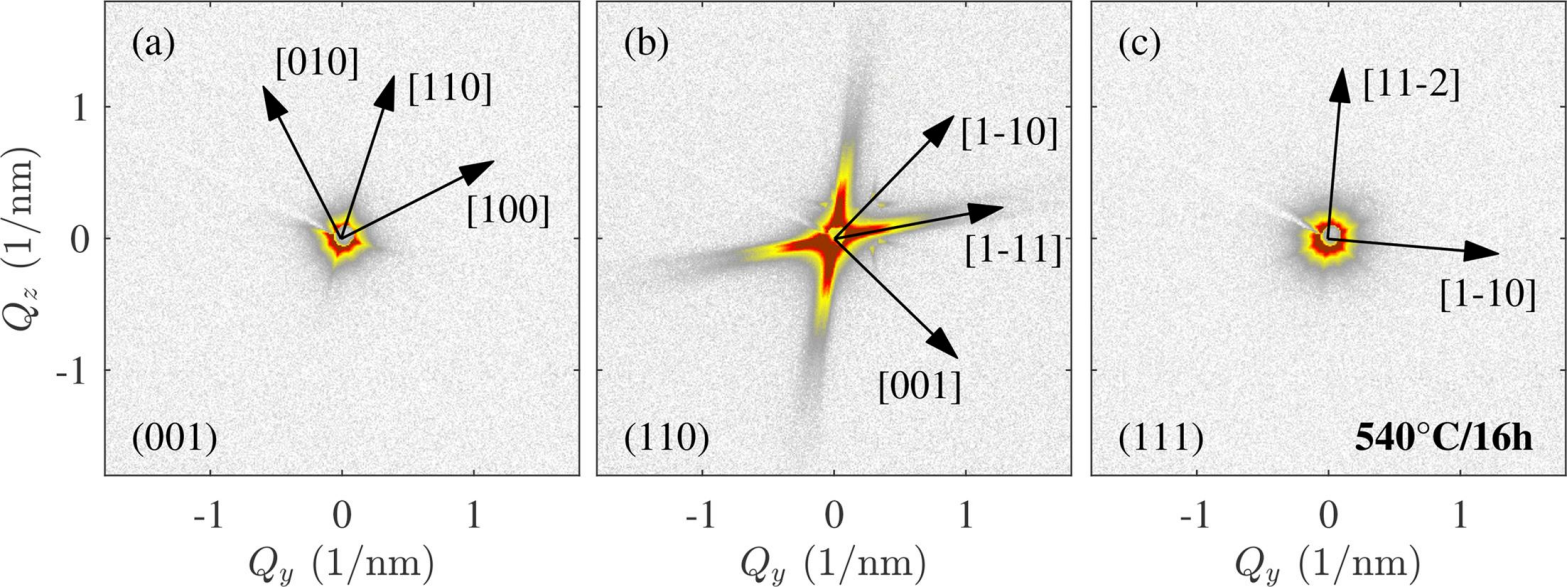
An example of small-angle X-ray scattering patterns arising from α lamellae in an aged LCB single crystal (540°C for 16 h). The plane of the b.c.c. β matrix which is perpendicular to the primary beam is indicated in the bottom-left corner of each detector image. The arrows show crystallographic directions determined from the Laue method. The colour scale is logarithmic and spans the range of three decades.

Fig. 1 ▸ shows SAXS patterns collected in three different orientations of the LCB single crystal aged for 16 h at 540°C, with the (001)_β_, (110)_β_ and (111)_β_ planes of the b.c.c. β matrix oriented perpendicular to the incident beam. The observed contrast in the SAXS images arises from variations in electronic density in the material (Feigin & Svergun, 1987 ▸). In our case, this corresponds to the different chemical composition of α particles and the β matrix. Namely, the α phase is depleted in the β-stabilizing elements, Mo and Fe, and richer in Ti and Al (Lütjering & Williams, 2007 ▸; Nag *et al.*, 2009*a* ▸). The detector images in Fig. 1 ▸ show distinct streaks of scattered intensity that arise from the shape of the α particles (*i.e.* long, thin lamellae) dispersed in the single-crystalline β matrix. SAXS data are a representation of the sample microstructure in the reciprocal space (more precisely, a Fourier transform of the shape of the particles); therefore, long streaks correspond to short dimensions of α laths in the real space and vice versa. Note that the symmetry of the streaks correlates with the orientation of the sample with respect to the primary beam: in the (001)_β_, (110)_β_ and (111)_β_ orientations, fourfold, twofold and sixfold symmetries are observed, respectively. This symmetry originates from the spatial orientation of α lamellae, as they grow in certain directions in the β grain.

In a recent article, we presented a detailed analysis of SAXS patterns from all ageing conditions, including fitting of the α lamella shape and orientation (Šmilauerová *et al.*, 2023 ▸). In this work, it was concluded that the habit plane of the α lamellae is close to but not exactly {111}_β_. The fact that the habit plane is not exactly {111}_β_ has consequences in the microstructure of the alloy – the apparent directions of α lamellae on the sample surface slightly fan out around four principal directions. If the habit plane was precisely {111}_β_, there would be exactly four apparent directions of the α laths. Furthermore, by fitting the SAXS patterns, it was determined that the longest α lamella axis lies approximately along one of the 〈110〉_β_ directions parallel to the habit plane.

**Figure 2:**
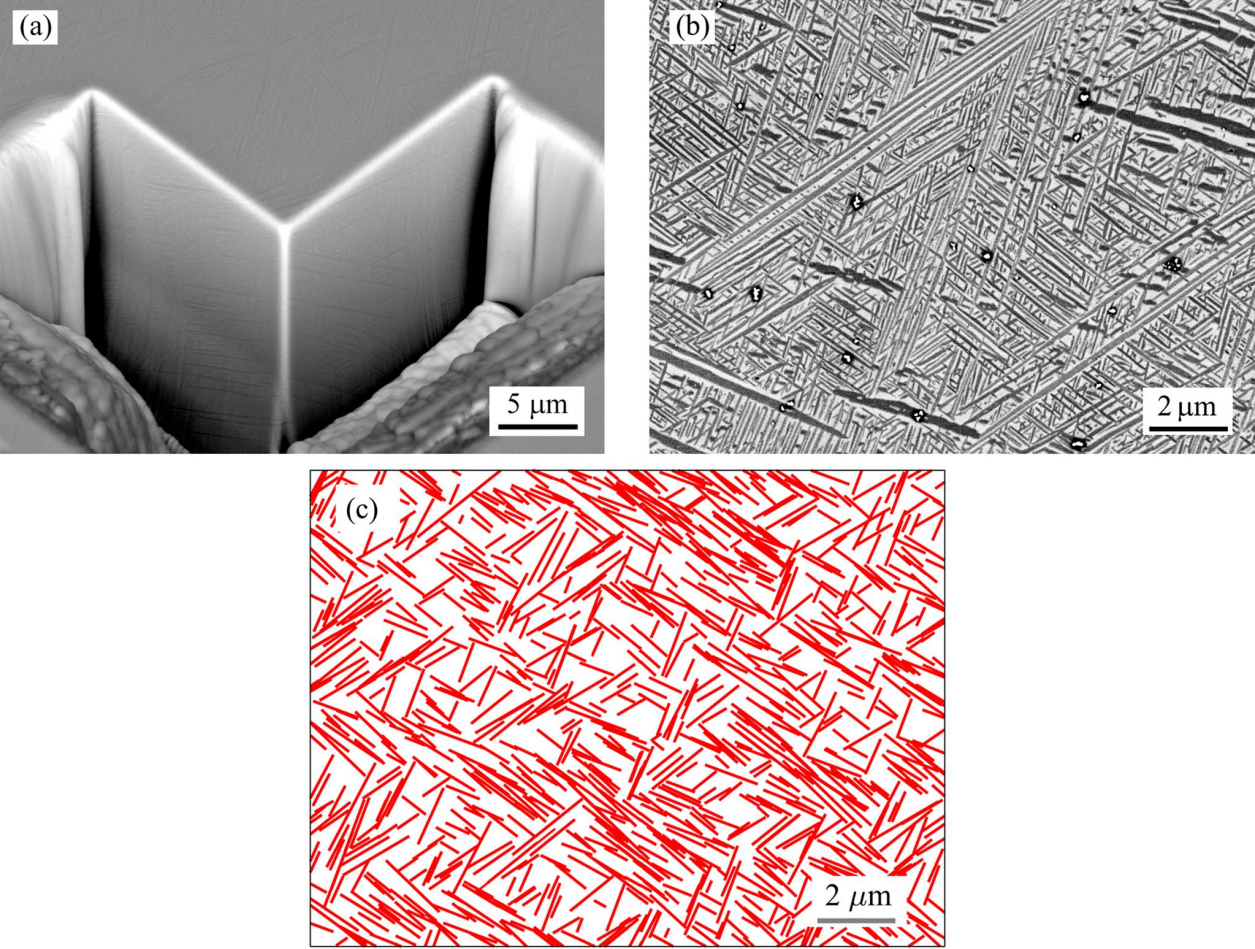
(*a*) SEM micrograph showing a part of a cube milled into the sample surface by FIB. (*b*) A detail of α phase lamellae on the top surface of the milled cube corner (backscattered electron contrast). The images in (*a*) and (*b*) were taken using accelerating voltages of 15 and 2 kV, respectively. (*c*) shows the result of a numerical simulation of the α lamellae using the orientations and sizes from the SAXS measurement.

To better understand the streak symmetries in the SAXS patterns and analyse the relationship between the spatial and crystallographic orientation of individual α variants, a detailed SEM analysis was performed employing FIB and EBSD. Using FIB, a part of a cube was milled into the surface of the sample [see Fig. 2 ▸(*a*)], which allowed us to directly observe α lamellae in three perpendicular planes. A detail of α phase morphology from the top surface of the cube corner is shown in Fig. 2 ▸(*b*). A basket-weave microstructure, typical for metastable β-titanium alloys aged in the α + β domain, is observed. From the SEM image, it can be seen that there are four principal apparent directions of α lamellae. However, these ‘directions’ are, in fact, cross sections of α platelets of various orientations in a 3D space with the surface of the sample and the actual orientation of the longest axis of each α lath may be different. The broad faces of α platelets with a common apparent direction lie on a similar plane; in other words, α lamellae with the same apparent direction share a similar habit plane. Šmilauerová *et al.* (2023 ▸) showed that each of the four principal apparent directions is actually composed of several similarly oriented directions and that this fact is a consequence of the α habit plane being close to but not exactly {111}_β_. As a result of the slight deviation of the α habit plane from {111}_β_, there are 24 different spatial orientations of α lamellae with respect to the β matrix (the b.c.c. β phase, belonging to the *O*_*h*_ point group, possesses the full cubic symmetry which includes 24 pure rotations without inversion).

The existence of four principal apparent α directions is general, and we believe this fact is not violated even in the triangular α arrangements [see *e.g.* Miyano *et al.* (2006 ▸)]. In these cases, three α variants combine to form a triangular pyramid. The fourth α orientation may be significantly suppressed, or the failure to observe it could be caused by the fact that these triangular shapes are often displayed looking along one of the 〈111〉_β_ directions. As a result, the α platelets with habit planes near the particular {111}_β_ plane will lie nearly parallel to the sample surface, appearing as unfocused smudges on SEM images taken using a ‘standard’ accelerating voltage. When the accelerating voltage is not significantly decreased, the signal comes from a relatively large volume beneath the sample surface. Therefore, the signal will contain information from both the thin α lamella and the β matrix that lies above or below it. In this configuration, α lamellae lying nearly parallel to the sample surface can easily be overlooked or disregarded as polishing artefacts. When a sufficiently low accelerating voltage is used [see Fig. 2 ▸(*b*) taken at 2 kV], even these α lamellae are clearly visible. At a higher accelerating voltage, α lamellae which lie nearly parallel to the sample surface [appearing as thicker ones in the low-energy image, Fig. 2 ▸(*b*), *e.g.* in the top-right and lower-left corners] would appear as unfocused, darker shadows.

Fig. 2 ▸(*c*) shows a simulation based on the lamella orientations and sizes determined from the SAXS data (the simulation procedure was described in detail in Section 2). Each line in Fig. 2 ▸(*c*) effectively represents an intersection of a simulated α lamella in three dimensions with an appropriately oriented sample surface. As explained by Šmilauerová *et al.* (2023 ▸), the SAXS experiment cannot resolve particles larger than approximately 1 µm. Consequently, the SAXS data represent only smaller α lamellae and the size distribution of α lamellae in the simulated image is more homogeneous than in the real microstructure. The simulation cannot obviously yield exact lamella positions as seen in the SEM image. However, the figures are qualitatively similar and show comparable apparent lamella directions, which confirms the validity of the SAXS results and provides a link between the indirect SAXS technique and the direct SEM observation.

**Figure 3:**
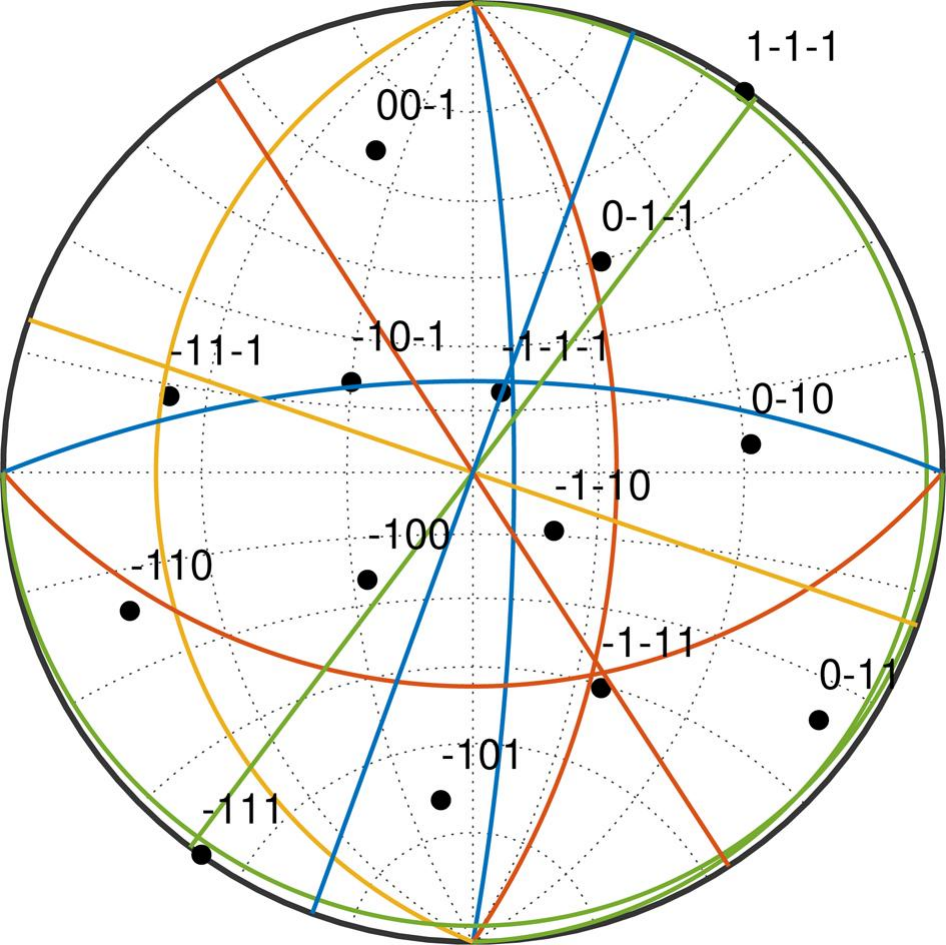
A stereographic projection of the β matrix orientation obtained by the Laue method. Coloured straight lines, vertical arcs and horizontal arcs represent the traces of planes normal to the apparent directions of α laths on the top, side and front face of the FIB-milled cube corner [Fig. 2 ▸(*a*)], respectively. The four different colours correspond to four apparent α lamella directions. A yellow horizontal arc is missing because the corresponding group of α laths exhibited only very short cross sections with the front surface of the cube in Fig. 2 ▸(*a*) and, therefore, the normal to the apparent α direction could not be determined accurately.

Using the knowledge of the orientation matrix of the sample obtained from the Laue method, a stereographic projection can be constructed (see Fig. 3 ▸). Black dots in Fig. 3 ▸ represent selected low-index β poles of the upper face of the cube corner in Fig. 2 ▸(*a*). The coloured lines represent the stereographic projections of normals to the longest apparent direction of α lamellae observed on the three perpendicular surfaces of the FIB-milled cube corner in Fig. 2 ▸. All normals to a given longest apparent direction of α laths lie on a common plane; therefore, the coloured lines in Fig. 3 ▸ are in fact traces of these planes in the stereographic projection. The straight coloured lines are the normals to the ‘directions’ of α laths (in the sense explained above) on the upper surface of the cube corner, while the vertical and horizontal arcs represent the normals to α lamellae on the side and front face of the cube, respectively. The colours of the arcs and straight lines represent the four apparent directions of the α lamellae.

The traces of the planes normal to a given apparent direction of α lamellae (*i.e.* coloured lines in Fig. 3 ▸ with the same colour) on the three faces of the FIB-milled cube corner [Fig. 2 ▸(*a*)] intersect near the {111}_β_ poles. From this fact we can conclude that the α lamellae grow approximately parallel to {111}_β_ planes. In other words, the broad face of an α lath, or its habit plane, is close to one of the {111}_β_ planes. This is consistent with the previous SAXS study of the spatial orientation of α platelets (Šmilauerová *et al.*, 2023 ▸). Note that the current means to determine the habit plane of the α lamellae, *i.e.* the combination of SEM and the knowledge of the crystal orientation, is not very precise; from these data we are only able to say that the habit plane is close to {111}_β_ but cannot index it properly. However, employing the EBSD technique, we are able to determine the orientation of the h.c.p. α lattice within an α lamella, which is not possible by SAXS and which, to the best of our knowledge, is missing from the literature.

**Figure 4:**
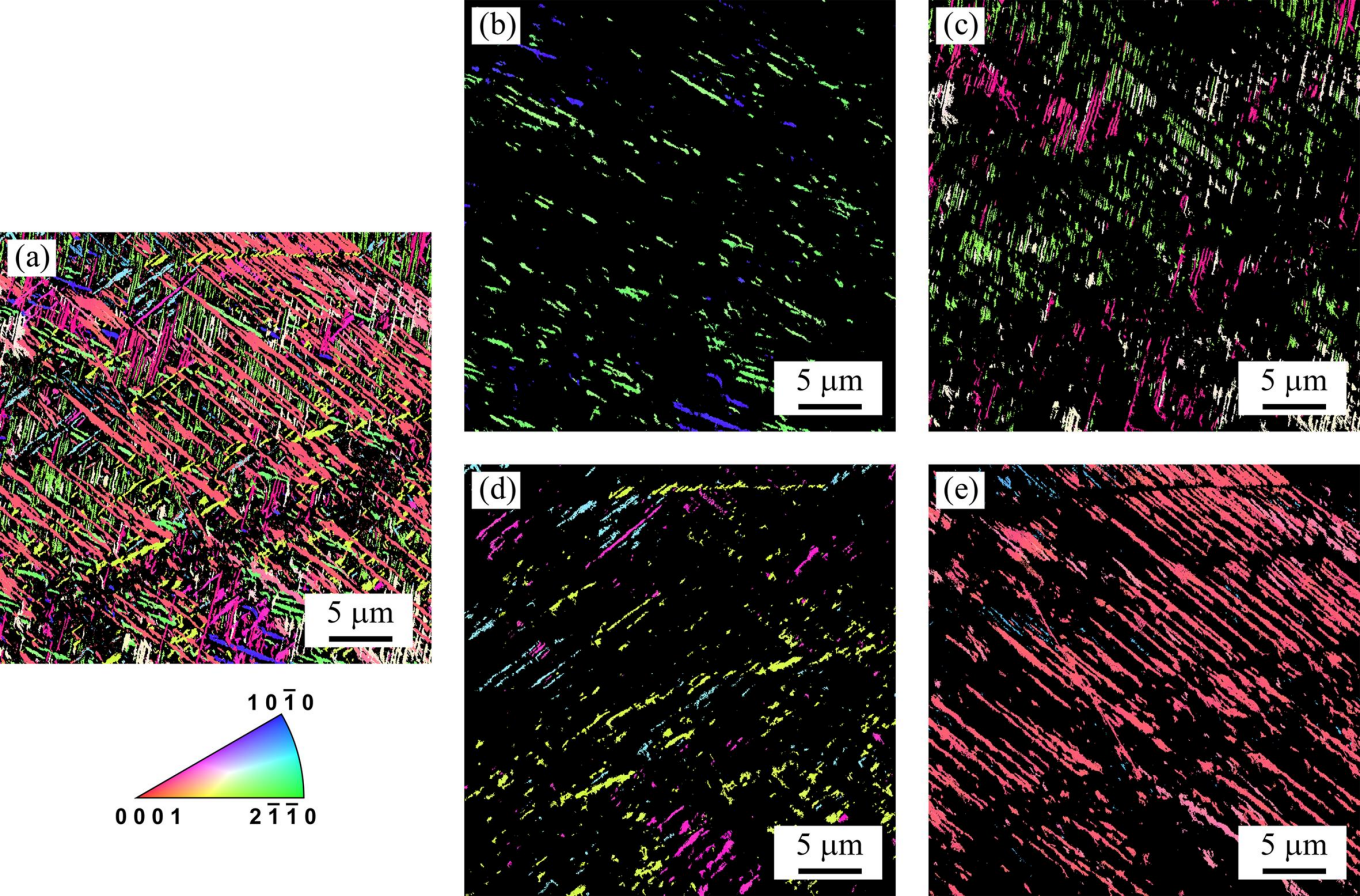
IPF maps showing (*a*) all 12 α variants on the top surface of the studied sample; (*b*)–(*e*) four groups of α lamellae, each containing three crystallographic variants sharing a common habit plane and, consequently, the same apparent direction on the cross section with the sample surface.

EBSD analysis was performed on a part of the polished sample surface (see Fig. 4 ▸). The region shown in Fig. 4 ▸ is a different place on the sample surface from the cube corner (Fig. 2 ▸); however, it represents the same sample with an identical orientation. The colours in the inverse pole figure maps (IPF maps) in Fig. 4 ▸ correspond to pixels indexed as the α phase; black pixels were indexed as the β phase. All 12 crystallographic variants of the α phase predicted by the Burgers OR [equation (1)] were observed in the studied sample [see Fig. 4 ▸(*a*)]. Figs. 4 ▸(*b*)–4 ▸(*e*) show the same region of the sample, but each of these four IPF maps shows three crystallographic variants of the α phase having the same apparent direction on the cross section with the sample surface. The α lamellae with the same apparent ‘direction’ in fact share a common habit plane (or more precisely a similarly oriented habit plane, as it is close to but not exactly {111}_β_), as discussed above. The groups of α variants in Figs. 4 ▸(*b*), 4 ▸(*c*), 4 ▸(*d*) and 4 ▸(*e*) correspond to the traces of the planes normal to apparent α lamella directions in Fig. 3 ▸ denoted by the blue, yellow, red and green lines, respectively.

Some of the observed crystallographic variants of the α phase seem to have a lower fraction than others, which is a consequence of the spatial orientation of the α laths – the variants which appear short or sparse [*e.g.* blue colours in Figs. 4 ▸(*d*) or 4 ▸(*e*)] may be just tilted with their longest direction nearly perpendicular to the polished surface of the sample, while the seemingly long and abundant [*e.g.* pink colour in Fig. 4 ▸(*e*)] ones have their longest axis nearly parallel to the sample surface. Additionally, α platelets whose habit plane is nearly parallel to the sample surface will appear to be sparse in an IPF map. This is because, for an EBSD analysis, a high accelerating voltage needs to be used (15 kV in our case). Consequently, the interaction volume is large and the detected signal then comes from both the thin α lamella (nearly parallel to the sample surface) and the β phase below and/or above it (this issue is analogous to the influence of electron energy on SEM images discussed above). In some cases, the majority of the interaction volume may lie below the α lamella. Such pixels may be indexed with a high confidence index as the β phase, although an SEM image taken at a low accelerating voltage would clearly show the presence of an α lamella.

Last but not least, there can be a considerable size distribution within each crystallographic variant of the α phase depending on the imaged region. α plates which nucleated first tend to be larger than those which nucleated later and whose growth was therefore limited by the already formed α lamellae.

For the reasons described above, it is not possible to draw any conclusions about the volume fraction of individual α variants from any IPF map. Even SEM images may be deceptive in this regard, as we showed in the discussion of the connection between the accelerating voltage and the visibility of some α lamella orientations. On the other hand, statistically relevant experimental techniques (*i.e.* those in which the signal is collected from a significantly larger sample volume), such as SAXS, can provide information on the relative fractions of individual α variants. From the SAXS experiment of Šmilauerová *et al.* (2023 ▸), it is known that the relative fractions of all α variants are essentially the same.

**Figure 5:**
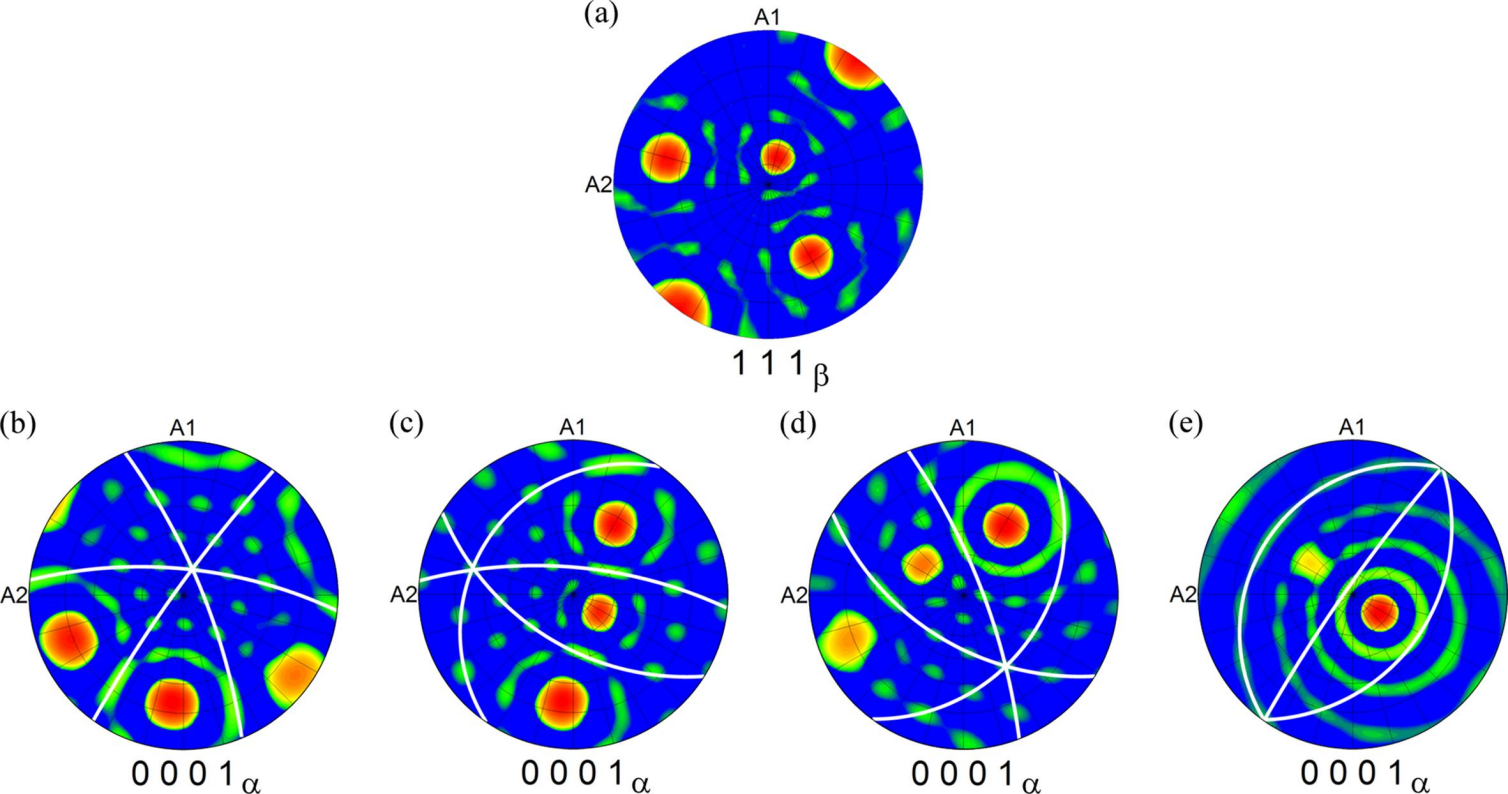
Pole figures showing (*a*) {111}_β_ poles of the β matrix. Each of the pole figures in (*b*), (*c*), (*d*) and (*e*) is a combination of three (0001)_α_ pole figures belonging to the three α variants which have the same apparent direction in Figs. 2 ▸ and 4 ▸. The white arcs in (*b*), (*c*), (*d*) and (*e*) are traces of the (0001)_α_ planes, *i.e.* all possible normals to the given [0001]_α_ direction. The intersections of the white arcs in each of the pole figures in (*b*), (*c*), (*d*) and (*e*) coincide with one of the {111}_β_ poles in (*a*), indicating that the basal planes of α variants with the same apparent direction are perpendicular to their common habit plane.

From the EBSD orientation data, we can demonstrate what the α variants with the same apparent direction have in common. Fig. 5 ▸(*a*) shows the {111}_β_ pole figure of the b.c.c. β phase, while each of the panels in Figs. 5 ▸(*b*), 5 ▸(*c*), 5 ▸(*d*) and 5 ▸(*e*) presents a combination of three (0001)_α_ pole figures with the same apparent orientation, *i.e.* the same (or more precisely, similar) habit plane. The groups of α variants shown in Figs. 5 ▸(*b*), 5 ▸(*c*), 5 ▸(*d*) and 5 ▸(*e*) are the same as those depicted in Figs. 4 ▸(*b*), 4 ▸(*c*), 4 ▸(*d*) and 4 ▸(*e*), respectively. The white arcs in Figs. 5 ▸(*b*), 5 ▸(*c*), 5 ▸(*d*) and 5 ▸(*e*) represent the traces of the (0001)_α_ planes. Note that, in each of the pole figures, the arcs intersect in one point, which also coincides with the position of one of the {111}_β_ poles in Fig. 5 ▸(*a*). From this fact it follows that the basal planes of the h.c.p. α lamellae sharing a common apparent direction are perpendicular to their common habit plane.

**Figure 6:**
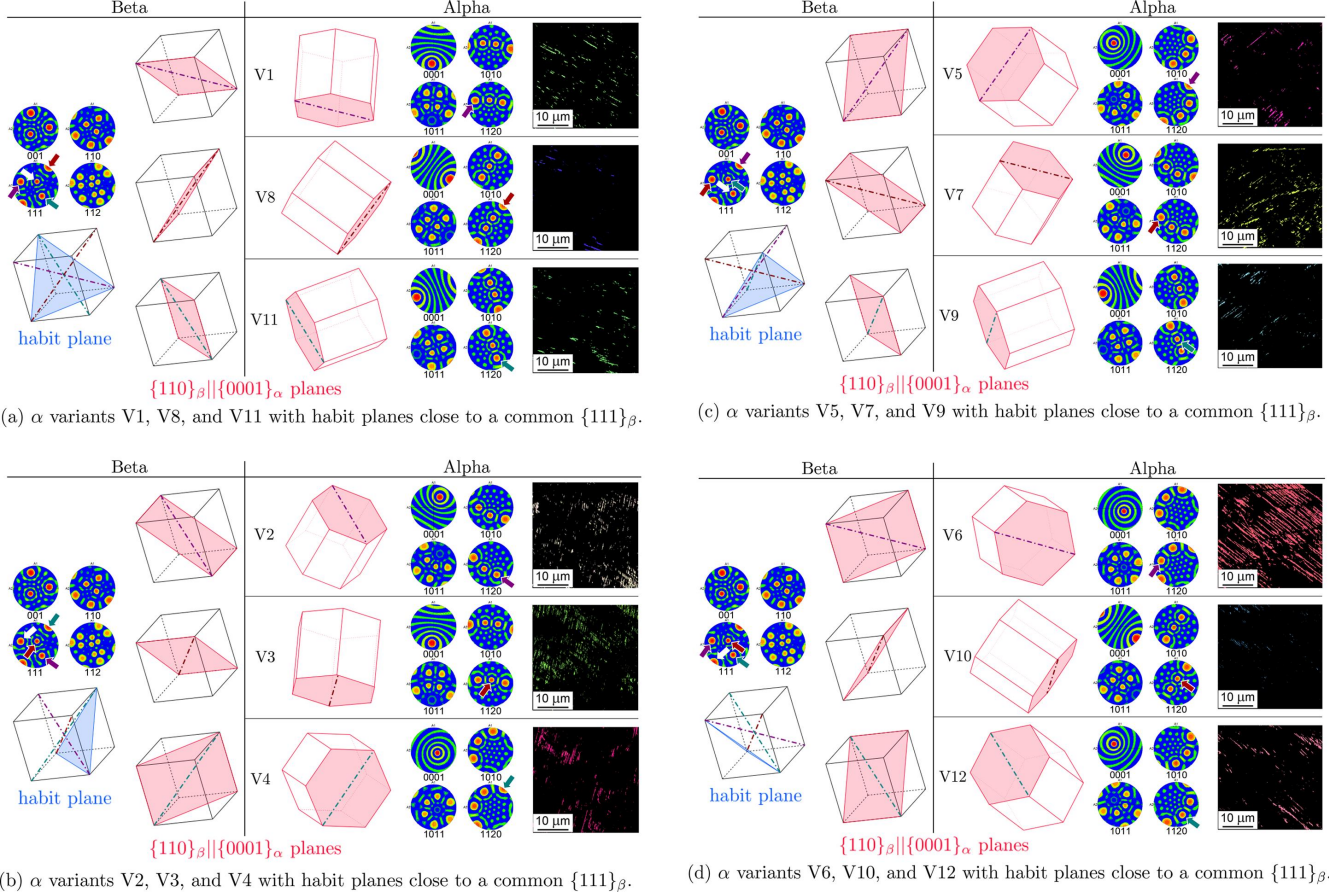
Summary of the 12 α variants separated into four groups according to the habit plane in the β matrix (denoted blue on the left side of the images). The red planes illustrate the mutual crystallographic orientation between β and α lattices (Burgers OR): the {110}_β_ planes to which the basal planes {0001}_α_ are parallel. The purple, red and green dash–dot lines show 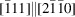, *i.e.* the second Burgers OR condition.

A detailed analysis of the EBSD data revealing the mutual OR of the b.c.c. β and h.c.p. α lattices for each of the 12 crystallographic variants of the α phase is presented in Fig. 6 ▸. Note that the angular resolution of the EBSD method is not as good as that of SAXS (Šmilauerová *et al.*, 2023 ▸). Therefore, we cannot detect the slight deviation of the α habit plane from {111}_β_ and, consequently, we cannot differentiate between the 24 possible spatial orientations of the α lamellae. Therefore, in the following, we will consider the α habit plane as {111}_β_ which yields 12 spatial orientations of the α lamellae due to the symmetry of the cubic b.c.c. β lattice.

Each of the subfigures (*a*), (*b*), (*c*) and (*d*) in Fig. 6 ▸ shows one type of {111}_β_ plane to which the α habit planes are close. The left part of the graphics in Fig. 6 ▸ displays the orientation of the cubic β lattice – the {001}_β_, {110}_β_, {111}_β_ and {112}_β_ pole figures accompanied by a correspondingly tilted model of the cubic cell (as seen when looking perpendicular to the sample surface). Similarly, the right-hand side of Fig. 6 ▸ shows the orientation of individual α variants, employing both the tilted model of the hexagonal cell and the pole figures 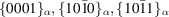 and 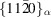. Finally, on the right of each figure, there are coloured IPF maps for each α variant [*cf*. Figs. 4 ▸(*b*), 4 ▸(*c*), 4 ▸(*d*), 4 ▸(*e*) where the three α variants with a common apparent direction and a common habit plane are presented in a combined IPF map].

The blue plane in the cubic cell in Fig. 6 ▸ shows the habit plane of the three variants of the α phase; the corresponding {111}_β_ pole (*cf*. Fig. 3 ▸) is marked in the pole figure by a white arrow. The planes shown in red are the {110}_β_-type planes to which the (0001)_α_ planes of the corresponding α variants (depicted in the hexagonal cells on the right-hand side of the figure) are parallel according to the Burgers OR [see equation (1)]. The red, green and violet dash–dot lines show the second part of the Burgers OR, *i.e.* the 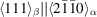 directions lying in the given {110}_β_||(0001)_α_ plane. Arrows of the same colours (red, green and violet) show the corresponding {111}_β_ and 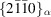 poles, making use of the fact that, in this specific case, the planes and directions having identical Miller indices (or Miller–Bravais for the hexagonal lattice) are perpendicular. Note that the highlighted {111}_β_ and 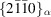 poles coincide in the respective β and α pole figures, which follows from the 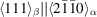 Burgers condition.

From Fig. 6 ▸, we can easily see that the basal planes of individual α variants, *i.e.* (0001)_α_||{110}_β_ shown in red, are perpendicular to the habit plane {111}_β_ (shown by the blue colour on the left-hand side of Fig. 6 ▸). This fact was already inferred from the pole figures in Fig. 5 ▸. Fig. 6 ▸ also shows the relationship between the orientation of the h.c.p. α lattice and the apparent α lamella orientation (in the cross section with the sample surface). Furthermore, Fig. 6 ▸ clearly demonstrates how both conditions of the Burgers OR are fulfilled for each α variant.

## 4. Indexing of the main directions and planes of an α lamella in the h.c.p. α coordinates

Until now, we have expressed the orientation of the α lath faces in the cubic coordinate system of the parent β phase. The (approximate) indices of the habit plane and the longest lamella direction with respect to the β phase are well known from the SAXS experiment of Šmilauerová *et al.* (2023 ▸); the habit plane is close to but not exactly {111}_β_, *i.e.* the shortest α lamella axis lies along one of the 〈111〉_β_ directions. The longest axis of an α lamella is roughly parallel to one of the three 〈110〉_β_ directions perpendicular to the given 〈111〉_β_ and the third lamella axis is along 〈112〉_β_, perpendicular to the remaining two axes. For the sake of clarity, we point out here the trivial fact that the pair of mutually perpendicular directions of 〈110〉_β_ and 〈112〉_β_ lie parallel to the given {111}_β_ habit plane. However, the SAXS technique does not provide us with any information on the orientation of the hexagonal α lattice in the α lamella; the indexing with respect to the β phase was possible only because the SAXS experiment was conducted on single crystals with a known orientation.

In this paper, we present a detailed study of the α lamellae using EBSD orientation analysis, which will allow us to approximately index the individual faces of the lamellae in the hexagonal α coordinate system. In the following, we will assume the Burgers OR between the β and α lattices [equation (1)], which is consistent with both the literature and our EBSD orientation data (see Fig. 6 ▸ and the accompanying text in Section 3).

**Figure 7:**
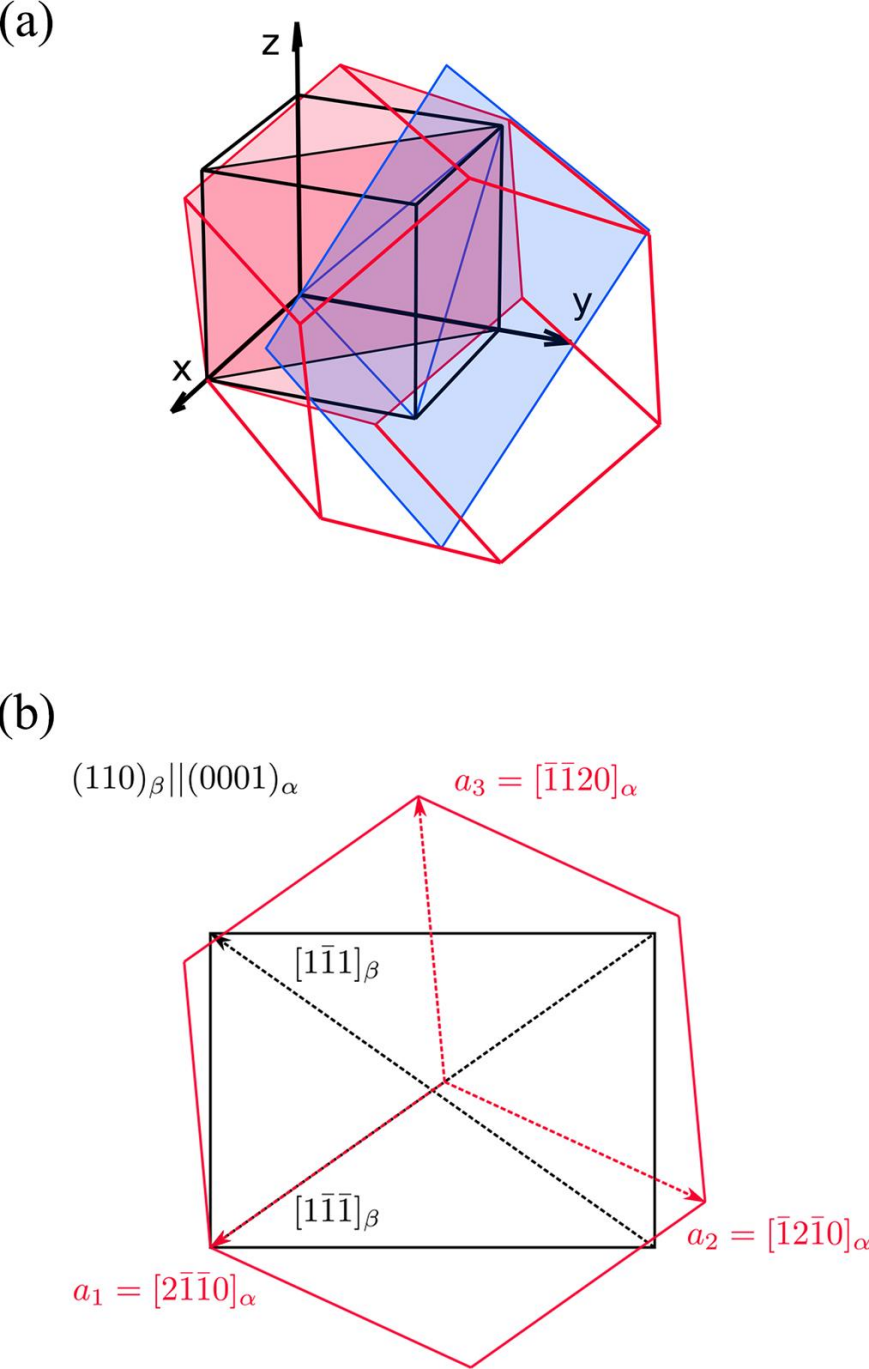
(*a*) A 3D representation of the mutual orientation of the cubic β phase (black) and one of the 12 α crystallographic variants (red). The blue plane is the habit plane of the α variant (the blue triangle indicates the intersection of this plane with the β cube). The red-coloured planes denote (110)_β_||(0001)_α_. (*b*) A perpendicular view of the (110)_β_||(0001)_α_ plane showing the indexing. The *a*_1_ axis of the hexagonal lattice was assumed to be the one aligned perfectly with the 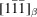 direction according to the Burgers OR. The *c* axis [0001]_α_ and the [110]_β_ direction point forward from the sketch.

Firstly, we know from the EBSD data that the {110}_β_ planes appearing in the Burgers OR, *i.e.* those to which the basal planes of the α lattices (0001)_α_ of lamellae sharing a common habit plane are parallel, are the three that are perpendicular to the specific {111}_β_ habit plane. For clarity, we will limit the following discussion to one particular α variant: namely the one whose basal plane is (0001)_α_||(110)_β_ with habit plane 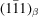 (see Fig. 7 ▸). However, one can easily generalize this discussion to all 12 crystallographic variants of the α phase (there are four {111}_β_ planes, each having three {110}_β_ planes perpendicular to it). The second Burgers condition for our selected α variant is 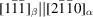, which corresponds to an orientation similar to variant V4 in Fig. 6 ▸(*b*). In the last condition, we assumed that the first coordinate axis of the hexagonal system, 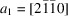, is the one perfectly aligned with the 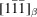 direction [see Fig. 7 ▸(*b*)]. The exact ratios of β and α axis lengths and the recalculation of directions and planes to the hexagonal system of the α phase depend on the lattice parameters of both the β and α phases. In Fig. 7 ▸ and the following, we assume 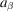 = 3.33 Å, 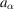 = 2.95 Å and 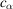 = 4.70 Å, which are average lattice parameters found in aged conditions of the LCB alloy (Šmilauerová *et al.*, 2017 ▸).

**Figure 8:**
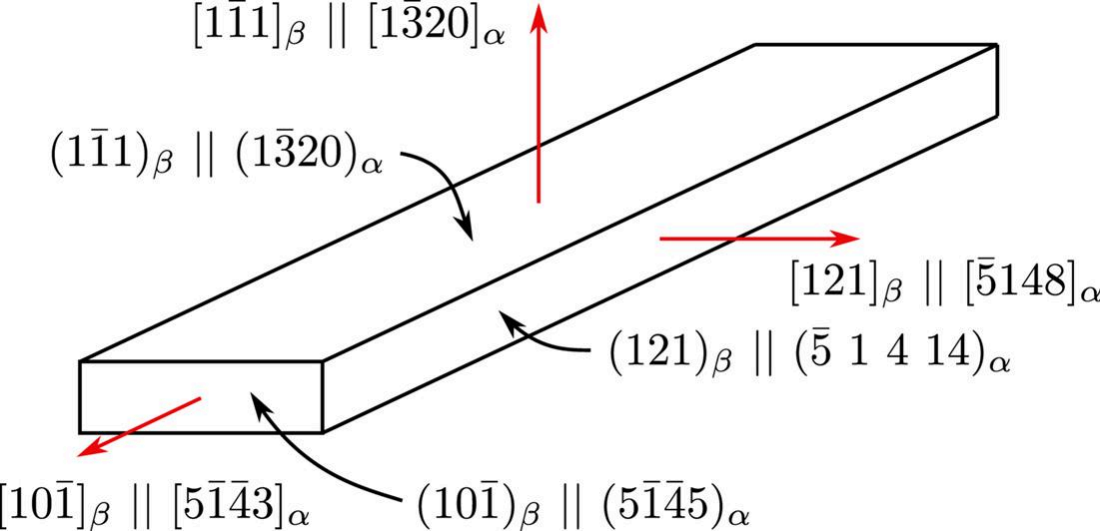
Schematics of an α platelet orientation. Only one of all possible α lath orientations is depicted (see the text for details). Note that the indices are only approximate, since the habit plane of the lamellae is close to, but not exactly, {111}_β_ and the indexing in the hexagonal coordinate system of the α phase also depends on the lattice parameters of both the β and α phases.

Knowing the mutual orientation of the α and β lattices and their lattice parameters, we may index the habit plane in the coordinate system of the α phase. From simple geometrical considerations, one can see that the habit plane is parallel to the *c* axis of the hexagonal α cell; consequently, the fourth index in the Miller–Bravais symbol of the plane must be zero. Furthermore, the habit plane intersects the *a*_1_ and *a*_2_ axes at 1 and −1/3, respectively. The indexing of the habit plane in the α coordinate system is therefore 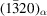. Consequently, the shortest α lamella dimension is along 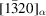. For a graphical interpretation of the indexing planes and directions of a selected variant of an α lamella, see Fig. 8 ▸.

The next step in the determination of the mutual orientation of the α lath and the α lattice is to find out which of the 〈110〉_β_ directions parallel to the 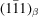 habit plane is the one corresponding to the longest α lamella direction. From the hexagonal prism orientations (or the pole figures) and apparent lengths of the corresponding α laths in Fig. 6 ▸, we can see that [110]_β_ which aligns with the [0001]_α_ direction cannot be the longest α lamella axis, since the α variants with the *c* axis nearly perpendicular to the sample surface appear significantly elongated on the cross section with the sample surface; see *e.g.* variants V4 and V6 in Figs. 6 ▸(*b*) and 6 ▸(*d*), respectively. If the longest α lath direction was nearly perpendicular to the sample surface, the apparent length of such a lath would be rather short. Here we mention that the apparent length of an α lamella is not determined entirely by its orientation with respect to the sample surface. Other factors, such as impingement on neighbouring α lamellae, also affect its true and apparent lengths. However, the area captured in the IPF maps is large enough to obtain relatively good statistics of the α lamellae, assuming that the individual variants nucleate randomly. Therefore, the longest α lath direction must be at an angle to the [110]_β_||[0001]_α_ direction. The two remaining 〈110〉_β_-type directions lying parallel to the 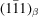 habit plane are [011]_β_ and 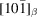. When the mutual orientation of the cubic β and the hexagonal α lattices and the lattice parameters of both phases (see above) are taken into account, the indexing of these two directions in the hexagonal system is 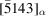 and 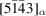, respectively (see Fig. 8 ▸ for one of the α variants). Note that this indexing is an approximation for two reasons: (i) the habit plane in the β phase is not exactly {111}_β_ and, consequently, the longest α lath axis may not also be perfectly aligned to one of the 〈110〉_β_ directions; and (ii) the recalculation from the cubic β coordinate system to the system connected to the hexagonal α depends on the particular lattice parameters of both phases.

Finally, the third α lamella axis (the intermediate dimension) must lie perpendicular to both the shortest and longest axes, *i.e.* to 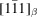 and 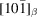, respectively, for the α variant shown in Fig. 8 ▸. This direction is [121]_β_ which corresponds to 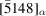. Again, this Miller–Bravais symbol is an approximation; more precise indexing would be 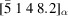.

Using the lattice parameters of the hexagonal α phase, we can also determine the indices of the planes of the α lath [for details see *e.g.* De Graef & McHenry (2012 ▸)]. We already stated that the habit plane in the hexagonal α coordinate system is 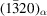. The smallest face of the α lamella, *i.e.* the face perpendicular to the longest α lath dimension, is approximately 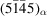. The remaining face of the α lamella is approximately 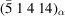. For a summary of the indexing for the selected variant of an α lamella, see Fig. 8 ▸. The indexing of directions and planes of the remaining α variants would follow the same logic; there would only be permutations and sign changes of the indices in the cubic system (assuming the chosen orientation of the hexagonal lattice is kept, *i.e.* the *a*_1_ axis is the one aligning perfectly to 〈111〉_β_ according to the Burgers OR, see Fig. 7 ▸).

## 5. Summary

Lamellae of the h.c.p. α phase were studied in an aged condition of a metastable β-Ti alloy, Timetal LCB (Ti–6.8 Mo–4.5 Fe–1.5 Al in wt%). A single crystal with a known orientation of the parent β matrix was used. The aim was to determine the spatial orientation of α lamellae within the β matrix and to index the main axes and planes of an α platelet not only in the cubic coordinates of the β phase but also in the hexagonal system of the α phase. In other words, we sought to determine the mutual orientation between the shape of an α lamella and the h.c.p. α crystallographic lattice inside it.

From small-angle X-ray scattering data, the α habit plane was established to be close to one of the four {111}_β_ planes. The longest and the intermediate axes of an α lath were determined to lie along one of the mutually perpendicular pairs of 〈110〉_β_ and 〈112〉_β_ directions parallel to the given habit plane, respectively.

SEM observation on three mutually perpendicular planes prepared by FIB milling of the studied sample surface confirmed that the habit plane is close to {111}_β_. Furthermore, it was observed that all α laths with a similar apparent direction on a sample surface also have a similar habit plane, close to a common {111}_β_. From this observation it follows that, since there are four different {111}_β_ planes, there are also four main apparent directions of α laths on any sample surface.

Information about the orientation of the α lattice in an α lamella was determined using EBSD in combination with the known mutual orientation of α and β lattices following from the Burgers OR. Considering one selected α variant (for the sake of clarity), the main planes and directions of an α lamella were determined as follows:


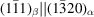
 is the habit plane,


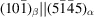
 is the smallest (growth) face,


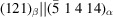
 is the side face,


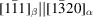
 is the shortest axis,


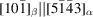
 is the longest axis and


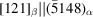
 is the intermediate axis of the α lath.
